# A method to integrate and classify normal distributions

**DOI:** 10.1167/jov.21.10.1

**Published:** 2021-09-01

**Authors:** Abhranil Das, Wilson S. Geisler

**Affiliations:** 1 Department of Physics, The University of Texas at Austin, Austin, TX, USA; 2 Department of Psychology, University of Texas at Austin, Austin, TX, USA; 3 Center for Perceptual Systems, University of Texas at Austin, Austin, TX, USA; 4 Center for Theoretical and Computational Neuroscience, University of Texas at Austin, Austin, TX, USA

**Keywords:** multivariate normal, integration, classification, signal detection theory, Bayesian ideal observer, vision

## Abstract

Univariate and multivariate normal probability distributions are widely used when modeling decisions under uncertainty. Computing the performance of such models requires integrating these distributions over specific domains, which can vary widely across models. Besides some special cases where these integrals are easy to calculate, there exist no general analytical expressions, standard numerical methods, or software for these integrals. Here we present mathematical results and open-source software that provide (a) the probability in any domain of a normal in any dimensions with any parameters; (b) the probability density, cumulative distribution, and inverse cumulative distribution of any function of a normal vector; (c) the classification errors among any number of normal distributions, the Bayes-optimal discriminability index, and relation to the receiver operating characteristic (ROC); (d) dimension reduction and visualizations for such problems; and (e) tests for how reliably these methods may be used on given data. We demonstrate these tools with vision research applications of detecting occluding objects in natural scenes and detecting camouflage.

## Introduction

The univariate or multivariate normal (henceforth called simply “normal”) is arguably the most important and widely used probability distribution. It is frequently used because various central-limit theorems guarantee that normal distributions will occur commonly in nature and because it is the simplest and most tractable distribution that allows arbitrary correlations between the variables.

Normal distributions form the basis of many theories and models in the natural and social sciences. For example, they are the foundation of Bayesian statistical decision/classification theories using Gaussian discriminant analysis (Ng, 2019), and are widely applied in diverse fields such as vision science, neuroscience, probabilistic planning in robotics, psychology, and economics. These theories specify optimal performance under uncertainty and are often used to provide a benchmark against which to evaluate the performance (behavior) of humans, other animals, neural circuits, or algorithms. They also serve as a starting point in developing other models/theories that describe suboptimal performance of agents.

To compute the performance predicted by such theories, it is necessary to integrate the normal distributions over specific domains. For example, a particularly common task in vision science is classification into two categories (e.g., detection and discrimination tasks). The predicted maximum accuracy in such tasks is determined by integrating normals over domains defined by a quadratic decision boundary (Green & Swets, 1966; Duda et al., 2012). Predicted accuracy of some of the possible suboptimal models is determined by integrating over other domains.

Except for some special cases (Ruben, 1960; Genz & Bretz, 2009) (e.g., multinormal probabilities in rectangular domains, such as when two normals have equal covariance and the optimal classification boundary is flat), there exists no general analytical expression for these integrals, and we must use numerical methods, such as integrating over a Cartesian grid, or Monte Carlo integration.

Since the normal distribution tails off infinitely outward, it is inefficient to numerically integrate it over a finite uniform Cartesian grid, which would be large and collect ever-reducing masses outward, yet omit some mass wherever the grid ends. Also, if the normal is elongated by unequal variances and strong covariances, or the integration domain is complex and noncontiguous, naive integration grids will waste resources in regions or directions that have low density or are outside the domain. One then needs to visually inspect and arduously hand-tailor the integration grid to fit the shape of each separate problem.

Monte Carlo integration involves sampling from the multinormal, then counting the fraction of samples in the integration domain. This does not have the above inefficiencies or problems but has other issues. Unlike grid integration, the desired precision cannot be specified but must be determined by measuring the spread across multiple runs. Also, when the probability in the integration domain is very small (e.g., to compute the classification error rate or discriminability $d^{'}$ for highly separated normal distributions), it cannot be reliably sampled without a large number of samples, which costs resource and time (see the performance benchmark section for a comparison).

Thus, there is no single analytical expression, numerical method, or standard software tool to quickly and accurately integrate arbitrary normals over arbitrary domains or to compute classification errors and the discriminability index $d^{'}$. Evaluating these quantities is often simplified by making the limiting assumption of equal variance. This impedes the quick testing, comparison, and optimization of models. Here we describe a mathematical method and accompanying software implementation that provides functions to (a) integrate normals with arbitrary means and covariances in any number of dimensions over arbitrary domains; (b) compute the probability density function (pdf), cumulative distribution function (cdf), and inverse cdf of any function of a multinormal variable (normal vector); and (c) compute the performance of classifying among any number of normals. This software is available as a MATLAB toolbox “Integrate and classify normal distributions,” and the source code is at github.com/abhranildas/IntClassNorm.

We first review and assimilate previous mathematical results into a generalized chi-squared method that can integrate arbitrary normals over quadratic domains. Then we present a novel ray-trace method to integrate arbitrary normals over any domain, and consequently to compute the distribution of any real-valued function of a normal vector. We describe how these results can be used to compute error rates (and other relevant quantities) for Bayes-optimal and custom classifiers, given arbitrary priors and outcome cost matrix. We then present some methods to reduce problems to fewer dimensions for analysis or visualization. Next, we provide a way to test whether directly measured samples from the actual distributions in a classification problem are close enough to normal to trust the computations from the toolbox. After describing the methods and software toolbox with examples, we demonstrate their accuracy and speed across a variety of problems. We show that for quadratic-domain problems, both the generalized chi-squared method and the ray-trace method are accurate, but vary in relative speed depending on the particular problem. Of course, for domains that are not quadratic, only the ray-trace method applies. Finally, we illustrate the methods with two applications from our laboratory: modeling detection of occluding targets in natural scenes, and detecting camouflage.

## Integrating the normal

### In quadratic domains: The generalized chi-square method

Integrating the normal in quadratic domains is important for computing the maximum possible classification accuracy. The problem is the following: given a column vector x∼N(μ,Σ), find the probability that

(1) $$q\left(x\right)=x^{'}Q_{2}x+q_{1}^{'}x+q_{0}>0.$$

(Here and henceforth, bold uppercase symbols represent matrices, bold lowercase symbols represent vectors, and regular lowercase symbols represent scalars.)

This can be viewed as the multidimensional integral of the normal probability over the domain q(x)>0 (that we call the “normal probability view”), or the single-dimensional integral of the probability of the scalar quadratic function q(x) of a normal vector, above 0 (the “function probability view”).

Note that x=Sz+μ, where z is standard multinormal, and the symmetric square root $S=\Sigma^{\frac{1}{2}}$ may be regarded as the multidimensional sd, since it linearly scales the normal (like σ in one dimension), and its eigenvectors and values are the axes of the 1 sd error ellipsoid. We first invert this transform to standardize the normal: $z=S^{-1}\left(x-\mu \right)$. This decorrelates or “whitens” the variables and transforms the integration domain to a different quadratic:

(2) $$\begin{array}{cc} \tilde{q}\left(z\right) & \;=z^{'}{\tilde{Q}}_{2}z+{\tilde{q}}_{1}^{'}z+{\tilde{q}}_{0}>0,\text{with}\\ {\tilde{Q}}_{2} & \;=SQ_{2}S,\\ {\tilde{q}}_{1} & \;=2SQ_{2}\mu +Sq_{1},\\ {\tilde{q}}_{0} & \;=q(\mu ). \end{array}$$

Now the problem is to find the probability of the standard normal z in this domain. If there is no quadratic term ${\tilde{Q}}_{2}$, $\tilde{q}\left(z\right)$ is normally distributed, the integration domain boundary is a flat, and the probability is $\Phi (\frac{{\tilde{q}}_{0}}{∥{\tilde{q}}_{1}∥})$, where Φ is the standard normal cdf (Ruben, 1960). Otherwise, say ${\tilde{Q}}_{2}={\mathrm{RDR}}^{'}$ is its eigen-decomposition, where R is orthogonal (i.e., a rotoreflection). So $y=R^{'}z$ is also standard normal, and in this space the quadratic is

$$\begin{array}{ccc} \;\;\;\;\;\;\;\;\;\;\;\;\;\;\;\;\;\;\;\;\;\;\;\;\hat{q}\left(y\right) & \;= & y^{'}Dy+b^{'}y+{\tilde{q}}_{0}\;\;\left(b=R^{'}{\tilde{q}}_{1}\right)\\ & \;= & \sum_{i}\left(D_{i}y_{i}^{2}+b_{i}y_{i}\right)+\sum_{i^{'}}b_{i^{'}}y_{i^{'}}+{\tilde{q}}_{0}\\ & & \;\;\text{(}i\;\text{and}\;i^{'}\;\text{index}\;\text{the}\;\text{nonzero}\;\text{and}\;\text{zero}\;\text{eigenvalues)}\\ & \;= & \sum_{i}D_{i}{\left(y_{i}+\frac{b_{i}}{2D_{i}}\right)}^{2}+\sum_{i^{'}}b_{i^{'}}y_{i^{'}}+{\tilde{q}}_{0}\\ & & -\;\sum_{i}{\left(\frac{b_{i}}{2D_{i}}\right)}^{2}\\ & \;= & \sum_{i}D_{i}\;\chi_{1,{\left(b_{i}/2D_{i}\right)}^{2}}^{'2}+x, \end{array}$$

a weighted sum of noncentral chi-square variables $\chi^{'2}$, each with 1 degree of freedom, and a normal variable x∼N(m,s). So this is a generalized chi-square variable ${\tilde{\chi }}_{w,k,\lambda ,m,s}^{2}$, where we merge the noncentral chi-squares with the same weights, so that the vector of their weights w are the *unique* nonzero eigenvalues among $D_{i}$, their degrees of freedom k are the numbers of times the eigenvalues occur and their noncentralities, and normal parameters are

$$\begin{array}{ccc} & & \lambda_{j}=\frac{1}{4w_{j}^{2}}\sum_{i:D_{i}=w_{j}}b_{i}^{2},\;m=q\left(\mu \right)-w.\lambda ,\\ & & s=\sqrt{\sum_{i^{'}}b_{i^{'}}^{2}}. \end{array}$$

The required probability, $p\left({\tilde{\chi }}^{2}>0\right)$, is now a 1d integral, computable using, say, Ruben's (1962) or Davies's (1973) methods. We use the MATLAB toolbox “Generalized chi-square distribution” that we developed (source code is at github.com/abhranildas/gx2), which can compute the generalized chi-square parameters corresponding to a quadratic form of a normal vector, its statistics, cdf (using three different methods), pdf, inverse cdf, and random numbers.

Previous software implements specific forms of this theory for particular quadratics such as ellipsoids (Genz & Bretz, 2009). The method described here correctly handles all quadratics (ellipsoids, hyperboloids, paraboloids, and degenerate conics) in all dimensions.

### In any domain: The ray-trace method

We present below our method to integrate the normal distribution in an arbitrary domain, which takes an entirely different approach than the generalized chi-square method. The overview of the method is as follows. We first standardize the normal to make it spherically symmetric, and then we integrate it in spherical polar coordinates, outward from the center. We first calculate the radial integral by sending “rays” from the center to trace out the integration domain in every direction, that is, determine the points where each ray crosses into and out of the domain (akin to the computer graphics method of ray-tracing, which traces light rays outward from the projection center to compute where it hits the different objects it has to render). By knowing these crossing points, we then calculate the probability amount on each ray. Then we add up these probabilities over all the angles. This method of breaking up the problem produces fast and accurate results to arbitrary tolerance for all problem shapes, without needing any manual adjustment.

#### Standard polar form

The problem is to find the probability that f(x)>0, where f(x) is a sufficiently general function (with a finite number of zeros in any direction within the integration span around the normal mean, i.e., without rare pathologies such as the Dirichlet function with infinite zeros in any interval). As before, we first standardize the space to obtain $\tilde{f}\left(z\right)=f\left(Sz+\mu \right)$. Then we switch to polar axis-angle coordinates z and n: any point z=zn, where the unit vector n denotes the angle of that point, and z is its coordinate along the axis in this direction. Then the integral can be written as

$$\begin{array}{c} \int_{\tilde{\Omega }}{\left(2\pi \right)}^{-\frac{k}{2}}e^{-\frac{z^{2}}{2}}dz=\int_{n}dn\underset{\text{axial}\;\text{integral}}{\underset{︸}{\int_{{\tilde{\Omega }}_{n}}{\left(2\pi \right)}^{-\frac{k}{2}}e^{-\frac{z^{2}}{2}}z^{k-1}dz}}. \end{array}$$

where $\tilde{\Omega }$ is the domain where $\tilde{f}\left(z\right)>0$, and ${\tilde{\Omega }}_{n}$ is its slice along the axis n, that is, the intervals along the axis where the axial domain function ${\tilde{f}}_{n}\left(z\right)=\tilde{f}\left(zn\right)>0$. This may be called the “standard polar form” of the integral. dn is the differential angle element (dθ in two-dimensional (2d), sinθdθdϕ in three-dimensional (3d), etc.).

#### Integration domain on a ray

**Figure 1.**
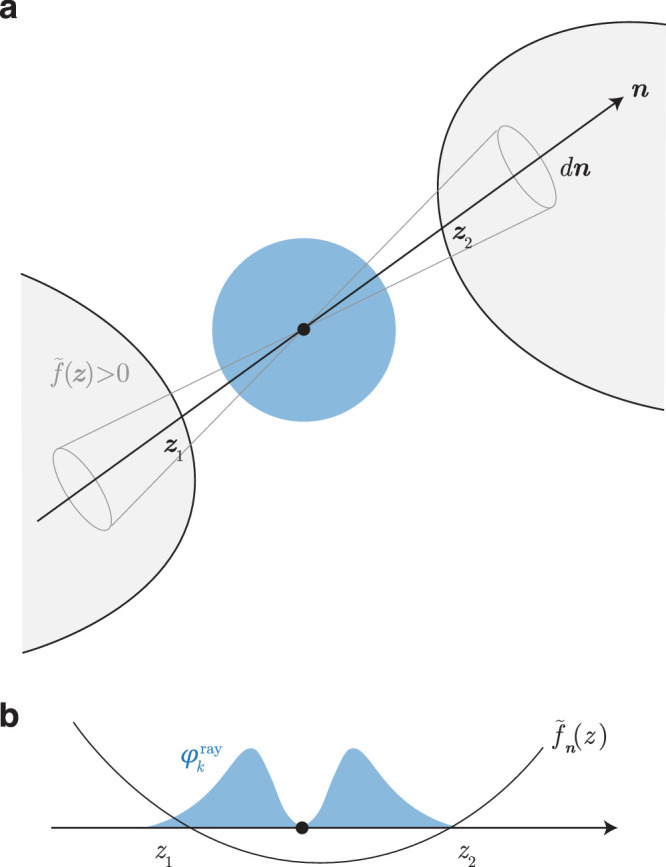
Method schematic. (**a**) Standard normal error ellipse is blue. Arrow indicates a ray from it at angle n in an angular slice dn, crossing the gray integration domain $\tilde{f}\left(z\right)>0$ at $z_{1}$ and $z_{2}$. (**b**) 1d slice of this picture along the ray. The standard normal density along a ray is blue. ${\tilde{f}}_{n}\left(z\right)$ is the slice of the domain function $\tilde{f}\left(z\right)$ along the ray, crossing 0 at $z_{1}$ and $z_{2}$.

First let us consider the axial integration along direction n. Imagine that we “trace” the integration domain with an axis through the origin in this direction (a bidirectional “ray” marked by the arrow in Figure 1a), that is, determine the part of this ray axis that is in the integration domain, defined by ${\tilde{f}}_{n}\left(z\right)>0$. For example, if the integration domain is a quadratic such as Equation 2, its 1d trace by the ray is given by

$$\begin{array}{cc} {\tilde{q}}_{n}\left(z\right)=\tilde{q}\left(zn\right) & \;=n^{'}{\tilde{Q}}_{2}nz^{2}+{\tilde{q}}_{1}^{'}nz+{\tilde{q}}_{0}\\ & \;={\tilde{q}}_{2}\left(n\right)\;z^{2}+{\tilde{q}}_{1}\left(n\right)\;z+{\tilde{q}}_{0}>0. \end{array}$$

This is a scalar quadratic domain in z that varies with the direction. Figure 1b is an example of such a domain. The ray domain function ${\tilde{f}}_{n}$ crosses 0 at $z_{1}$ and $z_{2}$, and the integration domain is below $z_{1}$ (which is negative) and above $z_{2}$.

Note that a sufficient description of such domains on an axis is to specify all the points at which the domain function crosses zero, and its overall sign, which determines which regions are within and which are outside the domain (so any overall scaling of the domain function does not matter). That is, we specify whether or not the beginning of the ray (at -∞) is inside the domain and all the points at which the ray crosses the domain. We denote the first by the initial sign $\psi \left(n\right)=\text{sign}({\tilde{f}}_{n}\left(-\infty \right))=1/-1/0$ if the ray begins inside/outside/grazing the integration domain. For a quadratic domain, for example:

$$\begin{array}{ccc} \psi (n) & \;= & \text{sign}\left({\tilde{q}}_{n}\left(-\infty \right)\right)\\ & \;= & \left\{\begin{array}{c} \text{sign}\left({\tilde{q}}_{2}\left(n\right)\right),\;\text{if}\;{\tilde{q}}_{2}\left(n\right)\neq 0,\\ -\text{sign}\left({\tilde{q}}_{1}\left(n\right)\right),\;\text{if}\;{\tilde{q}}_{2}\left(n\right)=0,\\ \text{sign}\left({\tilde{q}}_{0}\right),\;\text{if}\;{\tilde{q}}_{2}\left(n\right)={\tilde{q}}_{1}\left(n\right)=0. \end{array}\right. \end{array}$$

The crossing points are the zeros $z_{i}\left(n\right)$ of ${\tilde{f}}_{n}\left(z\right)=f\left(zSn+\mu \right)$ ($z_{i}n$ are then the boundary points in the full space). For a quadratic domain ${\tilde{q}}_{n}\left(z\right)$, these are simply its roots. For a general domain, the zeros are harder to compute. Chebyshev polynomial approximations (Trefethen, 2019) aim to find all zeros of a general function but can be slow. Other numerical algorithms can find all function zeros in an interval to arbitrary accuracy. We use such an algorithm to find the zeros of ${\tilde{f}}_{n}\left(z\right)$ within (-m,m). This amounts to ray-tracing f(x) within a Mahalanobis distance m of the normal. The error in the integral due to this approximation is therefore $<2\bar{\Phi }\left(m\right)$, where $\bar{\Phi }$ is the complementary cdf of the standard normal.

In Figure 1, the initial sign along the ray is 1, and $z_{1}$ and $z_{2}$ are the crossing points.

Most generally, this method can integrate in any domain for which we can return its “trace” (i.e., the initial sign and crossing points) along any ray n through any origin o. So if a domain is already supplied in the form of these “ray-trace” functions ψ(o,n) and $z_{i}\left(o,n\right)$, our method can readily integrate over it. For example, the ray-trace function of the line y=k in 2d returns $\psi =-\text{sign}(n_{y})$ and $z=\frac{k-o_{y}}{n_{y}}$. When supplied with quadratic domain coefficients, or a general implicit domain f(x)>0, the toolbox ray-traces it automatically under the hood. For an implicit domain, the numerical root-finding works only in a finite interval and is slower and may introduce small errors. So, if possible, a slightly faster and more accurate alternative to the implicit domain format is to directly construct its ray-trace function by hand.

#### Standard normal distribution on a ray

In order to integrate over piecewise intervals of z such as Figure 1b, we shall first calculate the semidefinite integral up to some z, then stitch them together over the intervals with the right signs.

Consider the probability in the angular slice dn below some negative z such as $z_{1}$ in Figure 1a. Note that the probability of a standard normal beyond some radius is given by the chi distribution. If $\Omega_{k}$ is the total angle in k dimensions (2 in 1d, 2π in 2d, 4π in 3d), and $F_{\chi_{k}}\left(x\right)$ is the cdf of the chi distribution with k degrees of freedom, we have

$$\begin{array}{c} \Omega_{k}\int_{-\infty }^{z<0}{\left(2\pi \right)}^{-\frac{k}{2}}e^{-\frac{z^{2}}{2}}z^{k-1}dz=1-F_{\chi_{k}}\left(|z|\right). \end{array}$$

So the probability in the angular slice dn below a negative z is $\left[1-F_{\chi_{k}}\left(|z|\right)\right]\frac{dn}{\Omega_{k}}$. Now, for the probability in the angular slice below a positive z (such as $z_{2}$), we need to add two probabilities: that in the finite cone from the origin to the point, which is $F_{\chi_{k}}\left(z\right)\frac{dn}{\Omega_{k}}$, and that in the entire semi-infinite cone on the negative side, which is $\frac{dn}{\Omega_{k}}$, to obtain $\left[1+F_{\chi_{k}}\left(z\right)\right]\frac{dn}{\Omega_{k}}$. Thus, the probability in an angular slice dn below a positive or negative z is $\left[1+\text{sign}\left(z\right)F_{\chi_{k}}\left(|z|\right)\right]\frac{dn}{\Omega_{k}}$. We normalize this by the total probability in the angular slice, $2\frac{dn}{\Omega_{k}}$, to define the distribution of the standard normal along a ray: $\Phi_{k}^{\text{ray}}\left(z\right)=\left[1+\text{sign}\left(z\right)F_{\chi_{k}}\left(|z|\right)\right]/2$. Its density is found by differentiating: $\phi_{k}^{\text{ray}}\left(z\right)=f_{\chi_{k}}\left(|z|\right)/2$, so it is simply the chi distribution symmetrically extended to negative numbers. Notice that $\phi_{1}^{\text{ray}}\left(z\right)=\phi \left(z\right)$, but in higher dimensions, it rises, then falls outward (Figure 1b), due to the opposing effects of the density falling but the volume of the angular slice growing outward. Since MATLAB does not yet incorporate the chi distribution, we instead define, in terms of the chi-square distribution, $\Phi_{k}^{\text{ray}}\left(z\right)=\left[1+\text{sign}\left(z\right)F_{\chi_{k}^{2}}\left(z^{2}\right)\right]/2$ and $\phi_{k}^{\text{ray}}\left(z\right)=\left|z\right|f_{\chi_{k}^{2}}\left(z^{2}\right)$.

#### Probability in an angular slice

We can now write the total probability in the angular slice of Figure 1 as the sum of terms accounting for the initial sign and each root. The total volume fraction of the double cone is $\frac{2dn}{\Omega_{k}}$. Now first consider only the initial sign and no roots. Then if the ray starts inside the domain (ψ=1), it stays inside, and the probability content is $\frac{2dn}{\Omega_{k}}$. If it begins and stays outside (ψ=-1), it is 0. And if it grazes the domain throughout (ψ=0), half of the angular volume is inside the domain and half is outside, so the probability is $\frac{dn}{\Omega_{k}}$. So without accounting for roots, the probability in general is $\frac{\psi (n)+1}{\Omega_{k}}$. To this we add, sequentially for each root, the probability from the root to ∞, signed according to whether we are entering or exiting the domain at that root. So we have, for Figure 1,

$$\begin{array}{c} dp\;\left(n\right)=\left[\frac{2}{\Omega_{k}}-\frac{2{\bar{\Phi }}_{k}^{\text{ray}}\left(z_{1}\right)}{\Omega_{k}}+\frac{2{\bar{\Phi }}_{k}^{\text{ray}}\left(z_{2}\right)}{\Omega_{k}}\right]dn. \end{array}$$

The sign of the first root term is always opposite to ψ, and subsequent signs alternate as we enter and leave the domain. In general, then, we can write

$$\begin{array}{cc} \;dp\;(n) & \;=\underset{\alpha (n)}{\underset{︸}{\left[\psi \left(n\right)+1+2\psi \left(n\right)\sum_{i}{\left(-1\right)}^{i}\;{\bar{\Phi }}_{k}^{\text{ray}}\left(z_{i}\left(n\right)\right)\right]}}\frac{dn}{\Omega_{k}} \end{array}$$

**Figure 2.**
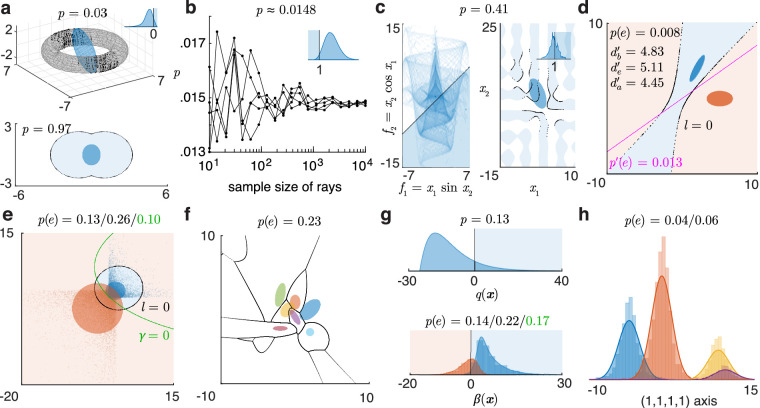
Toolbox outputs for some integration and classification problems. (**a**) Top: the probability of a 3d normal (blue shows 1 sd error ellipsoid) in an implicit toroidal domain $f_{t}\left(x\right)>0$. Black dots are boundary points within 3 sd traced by the ray method, across MATLAB's adaptive integration grid over angles. Inset: pdf of $f_{t}\left(x\right)$ and its integrated part (blue overlay). Bottom: integrating a 2d normal (blue error ellipse) in a domain built by the union of two circles. (**b**) Estimates of the 4d standard normal probability in the 4d polyhedral domain $f_{p}\left(x\right)=\sum_{i=1}^{4}\left|x_{i}\right|<1$ using the ray-trace method with Monte Carlo ray-sampling, across five runs, converging with growing sample size of rays. Inset: pdf of $f_{p}\left(x\right)$ and its integrated part. (**c**) Left: heat map of joint pdf of two functions of a 2d normal, to be integrated over the implicit domain $f_{1}-f_{2}>1$ (overlay). Right: corresponding integral of the normal over the domain $h\left(x\right)=x_{1}\sin x_{2}-x_{2}\cos x_{1}>1$ (blue regions), “traced” up to 3 sd (black dots). Inset: pdf of h(x) and its integrated part. (**d**) Classifying two 2d normals using the optimal boundary l (which yields the Bayes-optimal discriminability $d_{b}^{'}$) and a custom linear boundary. $d_{e}^{'}$ and $d_{a}^{'}$ are approximate discriminability indices. (**e**) Classification based on samples (dots) from non-normal distributions. Filled ellipses are error ellipses of fitted normals. γ is an optimized boundary between the samples. The three error rates are of the normals with l, of the samples with l, and of the samples with γ. (**f**) Classifying several 2d normals with arbitrary means and covariances. (**g**) Top: 1d projection of a 4d normal integral over a quadratic domain q(x)>0. Bottom: projection of the classification of two 4d normals based on samples, with unequal priors and unequal outcome values (correctly classifying the blue class is valued 4 times the red, and hence the optimal criterion is shifted), onto the axis of the Bayes decision variable β. Histograms and smooth curves are the projections of the samples and the fitted normals. The sample-optimized boundary γ=0 cannot be uniquely projected to this β axis. (**h**) Classification based on four 4d non-normal samples, with different priors and outcome values, projected on the axis along (1,1,1,1). The boundaries cannot be projected to this axis.

Thus, the axial integral is $\frac{\alpha (n)}{\Omega_{k}}$. The total probability $\frac{1}{\Omega_{k}}\int \alpha \left(n\right)\;dn$ can be computed, for up to 3d, by numerically integrating α(n) over a grid of angles spanning half the angular space (since we account for both directions of a ray), using any standard scheme. An adaptive grid can match the shape of the integration boundary (finer grid at angles where the boundary is sharply changing), and also set its fineness to evaluate the integral to a desired absolute or relative precision. Figure 2a, top, illustrates integrating a trivariate normal with arbitrary covariance in an implicitly defined toroidal domain $f_{t}\left(x\right)=a-{\left(b-\sqrt{x_{1}^{2}+x_{2}^{2}}\right)}^{2}-x_{3}^{2}>0$.

Beyond 3d, we can use Monte Carlo integration over the angles. We draw a sample of random numbers from the standard multinormal in those dimensions, then normalize their magnitudes, to get a uniform random sample of rays n, over which the expectation 〈α(n)〉/2 is the probability estimate. Figure 2b shows the computation of the 4d standard normal probability in the domain $f_{p}\left(x\right)=\sum_{i=1}^{4}\left|x_{i}\right|<1$, a 4d extension of a regular octahedron with plane faces meeting at sharp edges.

Since the algorithm already computes the boundary points over its angular integration grid, they may be stored for plotting and inspecting the boundary. Rather than an adaptive integration grid, though, boundaries are often best visualized over a uniform grid (uniform array of angles in 2D, or a Fibonacci sphere in 3D; Saff & Kuijlaars, 1997), which we can explicitly supply for this purpose.

#### Set operations on domains

Some applications require more complex integration or classification domains built using set operations (inversion/union/intersection) on simpler domains. With implicit domain formats, this is easy. For example, if $f_{A}\left(x\right)>0$ and $f_{B}\left(x\right)>0$ define two domains A and B, then $A^{c}$, A∩B, and $A\cup B^{c}$ are described by $-f_{A}\left(x\right)>0$, $\min(f_{A}\left(x\right),f_{B}\left(x\right))>0$, and $\max(f_{A}\left(x\right),-f_{B}\left(x\right))>0$, respectively. Figure 2a, bottom, illustrates integrating a 2d normal in a domain built by the union of two circles.

As we noted before, computations are faster and more accurate when domains are supplied in explicit ray-trace form than as implicit functions. The toolbox provides functions to convert quadratic and general implicit domains to ray-trace format, and functions to use set operations on these to build complex ray-trace domains. For example, when a domain is inverted, only the initial sign of a ray through it flips, and for the intersection of several domains, the initial sign of a ray is the minimum of its individual initial signs, and the roots are found by collecting those roots of each domain where every other domain is positive.

#### Probabilities of functions of a normal vector

We previously mentioned the equivalent “normal probability” and “function probability” views of conceptualizing a normal integral. So far, we have mostly used the normal probability view, seeing scalar functions f(x) as defining integral domains of the normal x. But in the function probability view, f(x) is instead seen as a mapping from the multidimensional variable x to a scalar, which can be considered a decision variable. Hence, integrating the normal in the multidimensional domain f(x)>0 corresponds to integrating the 1d pdf of the decision variable f(x) beyond 0. It is helpful to plot this 1d pdf, especially when there are too many dimensions of x to visualize the normal probability view.

Conversely, given any scalar function f(x) of a normal, its cdf, $F_{f}\left(c\right)=p\left(f\left(x\right)<c\right)$, is computed as the normal probability in the domain c-f(x)>0. Differentiating this gives us the pdf. (If it is a quadratic function, its generalized chi-square pdf can also be computed by convolving the constituent noncentral chi-square pdfs.) Figures 2a–c and g show 1d pdfs of functions computed in this way. Also, inverting the function cdf using a numerical root-finding method gives us its inverse cdf.

With these methods to obtain the pdf, cdf, and inverse cdf of functions of a normal vector, we can conveniently compute certain quantities. For example, if x and y are jointly normal with $\mu_{x}=1$, $\mu_{y}=2$, $\sigma_{x}=.1$, $\sigma_{y}=.2$, and $\rho_{xy}=.8$, we can compute the pdf, cdf, and inverse cdf of the function $x^{y}$ and determine, say, that its mean, median, and sd are respectively 1.03, 1, and 0.21.

The probability of a vector (multivalued) function of the normal, for example, $f\left(x\right)=[f_{1}\left(x\right)\;f_{2}\left(x\right)]$, in some f-domain (which may also be seen as the joint probability of two scalar functions) is again the normal probability in a corresponding x-domain. For example, the joint cdf $F_{f}\left(c_{1},c_{2}\right)$ is the function probability in an explicit domain: $p\left(f_{1}<c_{1},f_{2}<c_{2}\right)$ and can be computed as the normal probability in the intersection of the x-domains $f_{1}\left(x\right)<c_{1}$ and $f_{2}\left(x\right)<c_{2}$, that is, the domain $\min\left(c_{1}-f_{1}\left(x\right),c_{2}-f_{2}\left(x\right)\right)>0$. Numerically computing $\frac{\partial }{\partial c_{1}}\frac{\partial }{\partial c_{2}}F_{f}\left(c_{1},c_{2}\right)$ then gives the joint pdf of the vector function. Figure 2c, left, is an example of a joint pdf of two functions of a bivariate normal with μ=[-25] and $\Sigma =\left[\begin{array}{cc} 10 & -7\\ -7 & 10 \end{array}\right]$, computed in this way.

The probability of such a vector function in an implicit domain, that is, $p\left(g\left(f\right)>0\right)$, is computed as the normal probability in the implicit domain: $p\left(h\left(x\right)>0\right)$, where h=g∘f. Figure 2c illustrates the function probability and normal probability views of the implicit integral $p(h=x_{1}\sin x_{2}-x_{2}\cos x_{1}>1)$. The 83rd percentile of this function h (using the inverse cdf) is 4.87.

## Classifying normal samples

Suppose observations come from several normal distributions with parameters $\mu_{i},\Sigma_{i}$, and priors $p_{i}$, and the outcome values (rewards and penalties) of classifying them are represented in a matrix V: $v_{ij}$ is the value of classifying a sample from i as j.

If the true class is i, selecting i over others provides a relative value gain of $v_{i}:=v_{ii}-\sum_{j\neq i}v_{ij}$. Given a sample x, the expected value gain of deciding i is therefore $\langle v\left(i|x\right)\rangle =p\left(i|x\right)v_{i}=p\left(x|i\right)\;p_{i}v_{i}$. The Bayes-optimal decision is to assign each sample to the class that maximizes this expected value gain, or its log:

$$\begin{array}{ccc} \ln\langle v(i|x)\rangle & \;= & -\frac{1}{2}{\left(x-\mu_{i}\right)}^{'}\Sigma_{i}^{-1}\left(x-\mu_{i}\right)\\ & & +\;\ln\frac{p_{i}v_{i}}{\sqrt{|\Sigma_{i}{|\left(2\pi \right)}^{k}}}. \end{array}$$

When the outcome value is simply the correctness of classification, V=1 (so each $v_{i}=1$), then this quantity is the log posterior, lnp(i|x), and when priors are also equal, it is the log likelihood.

### Two normals

Suppose there are only two normal classes a and b. The Bayes-optimal decision rule is to pick A if (uppercase denotes the estimated classes)

(3) $$\begin{array}{ccc} \;\ln\frac{\langle v(A|x)\rangle }{\langle v(B|x)\rangle } & \;= & \beta \left(x\right)=x^{'}Q_{2}x+q_{1}^{'}x+q_{0}>0\text{,}\;\text{where}\\ Q_{2} & \;= & \frac{1}{2}\left(\Sigma_{b}^{-1}-\Sigma_{a}^{-1}\right),\\ q_{1} & \;= & \Sigma_{a}^{-1}\mu_{a}-\Sigma_{b}^{-1}\mu_{b},\\ q_{0} & \;= & \frac{1}{2}\left(\mu_{b}^{'}\Sigma_{b}^{-1}\mu_{b}-\mu_{a}^{'}\Sigma_{a}^{-1}\mu_{a}+\ln\frac{|\Sigma_{b}|}{|\Sigma_{a}|}\right)\\ & & +\ln\frac{p_{a}v_{a}}{p_{b}v_{b}}. \end{array}$$

This quadratic β(x) is the Bayes classifier, or the Bayes decision variable that, when compared to 0, maximizes expected gain.

When V=1, the Bayes decision variable is the log posterior ratio, and this decision rule minimizes overall error. The error rates of different types (i.e., true and false positives and negatives) are then the probabilities of the normals on either side of the quadratic boundary β(x)=0. These probabilities can be computed entirely numerically using the ray-trace method, or we can first arrive at mathematical expressions using the generalized chi-square method (as follows), which are then numerically evaluated. The overall error p(e) is the prior-weighted sum of the error rates of each normal.

Further, when priors are equal, the Bayes decision variable is the log likelihood ratio (of a vs. b), which can be called l(x).

#### Single-interval (yes/no) task

**Figure 3.**
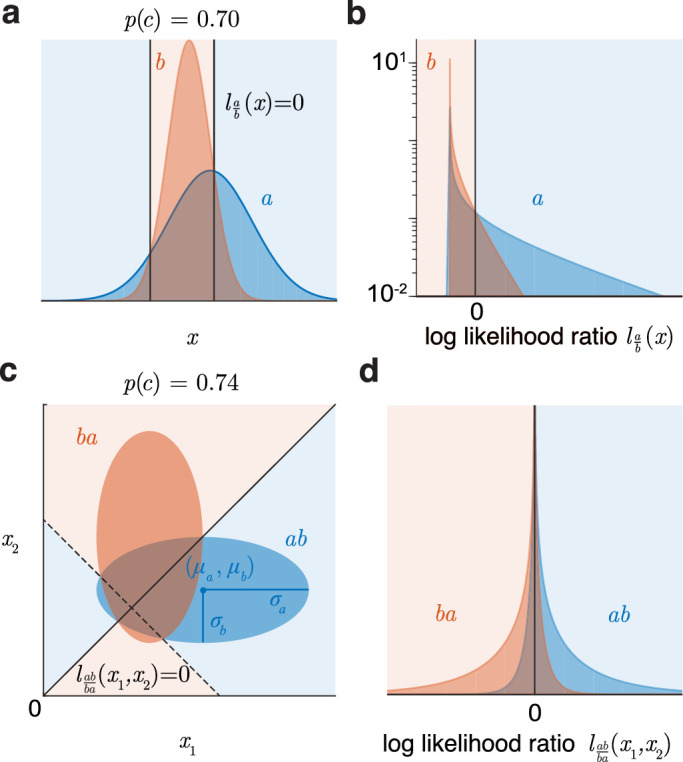
Binary yes/no and two-interval classification tasks. (**a**) Optimal yes/no decision between two unequal-variance 1d normal distributions. (**b**) The same task transformed to the log-likelihood ratio axis (log vertical axis for clarity). (**c**) Optimal two-interval discrimination between the same 1d normal distributions a and b is actually a discrimination between 2d normals ab and ba. (**d**) The task transformed to the log likelihood ratio axis.

Consider a yes/no task where the stimulus x comes from one of two equally likely 1d normals a and b with means $\mu_{a},\mu_{b}$ and sds $\sigma_{a}>\sigma_{b}$ (Figure 3a). The optimal decision (Equation 3) is to pick a if the Bayes decision variable (log-likelihood ratio of a vs. b) $l_{\frac{a}{b}}\left(x\right)>0$, that is, if

$$\begin{array}{c} {\left(\frac{x-\mu_{b}}{\sigma_{b}}\right)}^{2}-{\left(\frac{x-\mu_{a}}{\sigma_{a}}\right)}^{2}+2\ln\frac{\sigma_{b}}{\sigma_{a}}>0. \end{array}$$

$l_{\frac{a}{b}}\left(x\right)$ is a scaled and shifted 1 degree-of-freedom noncentral chi-square for each class (Figure 3b), and the Bayes error rates are

(4) $$\begin{array}{ccc} & & \;\;\;\;\;\;p\left(B|a\right)=p\left(\chi_{1,\sigma_{a}^{2}\lambda }^{'2}<\sigma_{b}^{2}c\right),\\ & & \;\;\;\;\;\;p\left(A|b\right)=p\left(\chi_{1,\sigma_{b}^{2}\lambda }^{'2}>\sigma_{a}^{2}c\right),\\ & & \;\;\;\;\;\;\text{where}\;\lambda ={\left(\frac{\mu_{a}-\mu_{b}}{\sigma_{a}^{2}-\sigma_{b}^{2}}\right)}^{2},\;c=\lambda +\frac{2\ln\frac{\sigma_{a}}{\sigma_{b}}}{\sigma_{a}^{2}-\sigma_{b}^{2}},\; \end{array}$$

and p(e) is their average.

#### Two-interval task

Now consider an equal-priors two-interval task, where two stimuli $x_{1}$ and $x_{2}$ come from each of the (general) distributions a and b. A decision rule commonly employed here is to check which stimulus is larger (Simpson & Fitter, 1973; Green, 2020). But note that the optimal strategy is to determine whether the tuple $x=(x_{1},x_{2})$ came from the joint distribution ab of independent a and b (in that order), or from ba (opposite order). To do this, we compute, given x, the log likelihood ratio $l_{\frac{ab}{ba}}$ of ab versus ba, which turns out to be simply related to the log-likelihood ratios $l_{\frac{a}{b}}$ for the individual stimuli in the single-interval task:

$$\begin{array}{ccc} \frac{p(ab|x)}{p(ba|x)} & \;= & \frac{p(x|ab)}{p(x|ba)}=\frac{p\left(x_{1}|a\right).p\left(x_{2}|b\right)}{p\left(x_{1}|b\right).p\left(x_{2}|a\right)}\\ & \;= & \frac{p\left(a|x_{1}\right).p\left(b|x_{2}\right)}{p\left(b|x_{1}\right).p\left(a|x_{2}\right)}\\ & \;\Rightarrow & l_{\frac{ab}{ba}}\left(x_{1},x_{2}\right)=l_{\frac{a}{b}}\left(x_{1}\right)-l_{\frac{a}{b}}\left(x_{2}\right). \end{array}$$

The optimal rule is to pick ab if $l_{\frac{ab}{ba}}\left(x_{1},x_{2}\right)>0$, that is, if $l_{\frac{a}{b}}\left(x_{1}\right)>l_{\frac{a}{b}}\left(x_{2}\right)$. This is the familiar decision rule (Green & Swets, 1966): The observer gets a log-likelihood ratio from each distribution for the single-interval task (e.g., Figure 3b) and picks the larger likelihood ratio (not the larger stimulus).

When a and b are normals, ab is the 2d normal with mean $\mu_{ab}=\left(\mu_{a},\mu_{b}\right)$ and sd $S_{ab}=\text{diag}\left(\sigma_{a},\sigma_{b}\right)$, and ba is its its flipped version (Figure 3c). The optimal decision rule (Equation 3) boils down to selecting ab when

$$\begin{array}{ccc} & & \left(\sigma_{a}^{2}-\sigma_{b}^{2}\right)\left(x_{1}^{2}-x_{2}^{2}\right)\\ & & \;+\;2\left(\mu_{a}\sigma_{b}^{2}-\mu_{b}\sigma_{a}^{2}\right)\left(x_{1}-x_{2}\right)>0. \end{array}$$

When $\sigma_{a}=\sigma_{b}$, this is the usual condition of whether $x_{1}>x_{2}$. But when $\sigma_{a}\neq \sigma_{b}$, this optimal decision boundary comprises two perpendicular lines, solid and dashed (Figure 3c). The $x_{1}>x_{2}$ criterion is to use only the solid boundary, which is suboptimal.

The minimum error rate p(e) is the probability that the difference distribution of the two categories of Figure 3b exceeds 0. $l_{\frac{ab}{ba}}$ is the difference of scaled and shifted noncentral chi-squares $l_{\frac{a}{b}}$, so has generalized chi-square distributions for each category (Figure 3d), and we can calculate that $p\left(e\right)=p\left({\tilde{\chi }}_{w,k,\lambda ,0,0}^{2}\right)<0$, where

$$\begin{array}{c} \;\;\;\;\;\;\;\;\;\;\;\;\;\;w=\left[\begin{array}{cc} \sigma_{a}^{2} & -\sigma_{b}^{2} \end{array}\right],\;k=\left[\begin{array}{cc} 1 & 1 \end{array}\right],\;\lambda =\frac{\mu_{a}-\mu_{b}}{\sigma_{a}^{2}-\sigma_{b}^{2}}\left[\begin{array}{cc} \sigma_{a}^{2} & \sigma_{b}^{2} \end{array}\right]. \end{array}$$

If the two stimuli themselves arise from k-dimensional normals $N(\mu_{a},\Sigma_{a})$ and $N(\mu_{b},\Sigma_{b})$, then the optimal discrimination is between 2k-dimensional normals ab and ba, whose means are the concatenations of $\mu_{a}$ and $\mu_{b}$, and covariances are the block-diagonal concatenations of $\Sigma_{a}$ and $\Sigma_{b}$, in opposite order to each other.

#### m -interval task

Consider the m-interval (m-alternative forced choice) task with m stimuli, one from the signal distribution $N(\mu_{a},\sigma_{a})$ and the rest from $N(\mu_{b},\sigma_{b})$. Following previous reasoning, the probability of the ith stimulus being the signal is an m-d normal, with mean vector whose ith entry is $\mu_{a}$ and the rest are $\mu_{b}$, and diagonal sd matrix whose ith entry is $\sigma_{a}$ and the rest are $\sigma_{b}$. The part of this log-likelihood that varies across i is:

$$\begin{array}{ccc} & & -\sum_{j\neq i}{\left(\frac{x_{j}-\mu_{b}}{\sigma_{b}}\right)}^{2}-{\left(\frac{x_{i}-\mu_{a}}{\sigma_{a}}\right)}^{2}\\ & & \;=-\underset{\text{constant}}{\underset{︸}{\sum_{j}{\left(\frac{x_{j}-\mu_{b}}{\sigma_{b}}\right)}^{2}}}\\ & & \;+\underset{\text{varies}\;\text{with}\;\text{i}}{\underset{︸}{{\left(\frac{x_{i}-\mu_{b}}{\sigma_{b}}\right)}^{2}-{\left(\frac{x_{i}-\mu_{a}}{\sigma_{a}}\right)}^{2}}}. \end{array}$$

The optimal response is to pick the m-d normal with highest likelihood, that is, pick the $x_{i}$ with the largest value of the second term above, i.e. with the largest log-likelihood ratio l of a versus b, which is the familiar rule (Green & Swets, 1966).

Analogous to Wickens (2002), eq. 6.19, the maximum accuracy is then given by:

$$\begin{array}{c} p\left(c\right)=\int_{-\infty }^{\infty }F_{b}^{m-1}\left(l\right)\;f_{a}\left(l\right)\;dl \end{array}$$

where $f_{a}$ and $F_{b}$ are the pdf and cdf of l under a and b, which are known, so this can be evaluated numerically. For example, for two, three, and four intervals with the parameters of Figure 3a, the accuracy is 0.74 (Figure 3c), 0.64, and 0.58 (see example in the getting started guide for the toolbox). When the variances are equal, these computed accuracies match Table 6.1 of Wickens (2002).

#### Discriminability index

Bayesian classifiers are often used to model behavioral (or neural) performance in binary classification. Within the Bayesian modeling framework, it is possible to estimate, from the pattern of errors, the separation (or overlap) of the decision variable distributions for the two categories, independent of the decision criterion (which may differ from the optimal value of zero). The discriminability index $d^{'}$ measures this separation. If the two underlying distributions are equal-variance univariate normals a and b, then $d^{'}=\left|\mu_{a}-\mu_{b}\right|/\sigma$, and if they are multivariate with equal covariance matrices, then it is their Mahalanobis distance: $d^{'}=\sqrt{{\left(\mu_{a}-\mu_{b}\right)}^{'}\Sigma^{-1}\left(\mu_{a}-\mu_{b}\right)}=∥S^{-1}\left(\mu_{a}-\mu_{b}\right)∥=∥\mu_{a}-\mu_{b}∥/\sigma_{\mu }$, where $\sigma_{\mu }=1/∥S^{-1}\mu ∥$ is the 1d slice of the sd along the unit vector μ through the means, that is, the multidimensional $d^{'}$ equals the $d^{'}$ along the 1d slice through the means.

For unequal variances, there exist several contending discriminability indices (Wickens, 2002; Chaddha & Marcus, 1968; Simpson & Fitter, 1973). A common one is Simpson and Fitter's (1973) $d_{a}^{'}=\left|\mu_{a}-\mu_{b}\right|/\sigma_{\text{rms}}$, extended to general dimensions as the Mahalanobis distance using the pooled covariance, that is, with $S_{\text{rms}}={\left[\left(\Sigma_{a}+\Sigma_{b}\right)/2\right]}^{\frac{1}{2}}$ as the common sd (Paranjpe & Gore 1994). Another index is Egan and Clarke's (1962) $d_{e}^{'}=\left|\mu_{a}-\mu_{b}\right|/\sigma_{\text{avg}}$, which we here extend to general dimensions using $S_{\text{avg}}=\left(S_{a}+S_{b}\right)/2$.

These unequal-covariance measures are simple approximations that do not describe the exact separation between the distributions. However, our methods can be used to define a discriminability index that exactly describes the separation between two arbitrary distributions (even non-normal). First, we determine the minimum possible (Bayes) errors when V=1 and priors are equal. In terms of the distributions of the log-likelihood ratios, these are

$$\begin{array}{cc} p(B|a) & \;=\int_{-\infty }^{0}f_{a}\left(l\right)\;dl=F_{a}\left(0\right),\\ p(A|b) & \;=\int_{0}^{\infty }f_{b}\left(l\right)\;dl=1-F_{b}\left(0\right). \end{array}$$

(For 1d normals, these are given by Equation 4). The overall Bayes error p(e) is the average of these two and is the amount of overlap of the two distributions (e.g., the overlap area in Figure 3a). We now define the *Bayes discriminability index* as the equal-variance index that corresponds to this same Bayes error, that is, the separation between two unit variance normals that have the same overlap as the two distributions, which comes out to be twice the z-score of the maximum accuracy:

$$\begin{array}{cc} \;\;\;\;\;\;\;\;\;\;\;\;\;d_{b}^{'} & \;=-2Z(\text{Bayes}\;\text{error}\;\text{/}\;\text{overlap}\;\text{fraction}\;p(e))\\ \;\;\;\;\;\;\;\;\;\;\;\; & \;=2Z(\text{best}\;\text{accuracy}\;\text{/}\;\text{nonoverlapping}\;\text{fraction}\;p(c)) \end{array}$$

This index is the best possible discriminability, that is, by an ideal observer. It extends to all cases as a smooth function of the layout and shapes of the distributions and reduces to $d^{'}$ for equal variance/covariance normals.

$d_{b}^{'}$ is a positive-definite statistical distance measure that is free of assumptions about the distributions, like the Kullback–Leibler divergence $D_{\text{KL}}\left(a,b\right)$, which is the expected log-likelihood ratio l under the a distribution (mean of the blue distributions in Figures 3b and d). $D_{\text{KL}}\left(a,b\right)$ is asymmetric, whereas $d_{b}^{'}\left(a,b\right)$ is symmetric for the two distributions. However, $d_{b}^{'}$ does not satisfy the triangle inequality. For example, consider three equal-width, consecutively overlapping uniform distributions: a over [0,3], b over [2,5], and c over [4,7]. b overlaps with a and c: $d_{b}^{'}\left(a,b\right)=d_{b}^{'}\left(b,c\right)=2Z\left(2/3\right)$, but a and c do not overlap: $d_{b}^{'}\left(a,c\right)=\infty ≮d_{b}^{'}\left(a,b\right)+d_{b}^{'}\left(b,c\right)$.

**Figure 4.**
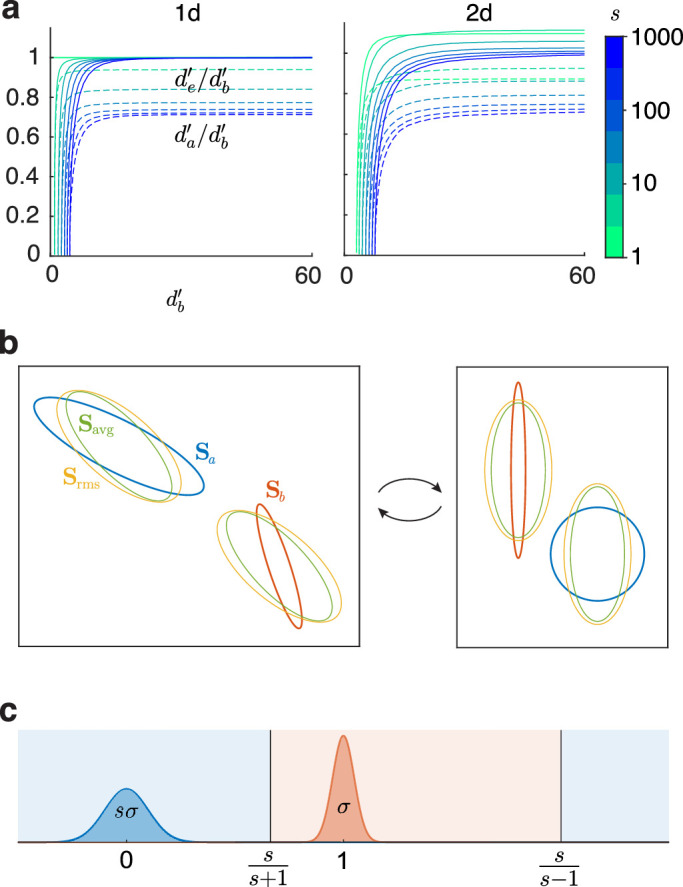
Comparing discriminability indices. (**a**) Plots of existing indices $d_{a}^{'}$ and $d_{e}^{'}$ as fractions of the Bayes index $d_{b}^{'}$, with increasing separation between two 1d and two 2d normals, for different ratios s of their sds. (**b**) Left: two normals with 1 sd error ellipses corresponding to their sd matrices $S_{a}$ and $S_{b}$, and their average and root-mean-square (rms) sd matrices. Right: the space has been linearly transformed, so that a is now standard normal, and b is aligned with the coordinate axes. (**c**) Discriminating two highly separated 1d normals.

In Figure 4a, we compare $d_{b}^{'}$ with $d_{a}^{'}$ and $d_{e}^{'}$ for different mean-separations and sd ratios of two normals, in 1d and 2d. We first take two 1d normals and increase their discriminability by equally shrinking their sds while maintaining their ratio $\sigma_{a}/\sigma_{b}=s$, that is, effectively separating the means. We repeat this by starting with two 2d normals with different sd matrices, one of them scaled by different values s each time, then shrink them equally.

Extending previous findings (Simpson & Fitter, 1973), we see that in 1d (Figure 4a left), $d_{a}^{'}\leq d_{e}^{'}\leq d_{b}^{'}$. Thus, $d_{a}^{'}$ and $d_{e}^{'}$ underestimate the optimal discriminability of normal distributions. The worst case is when the means are equal, so $d_{a}^{'}=d_{e}^{'}=0$, but $d_{b}^{'}$ is positive, since unequal variances still provide discriminability.

Now consider the opposite end, where large mean separation has a much greater effect on discriminability than sd ratios. Even here, the underestimate by $d_{a}^{'}$ persists and worsens as the sds become more unequal, reaching nearly 30% in the worst case. $d_{e}^{'}$ is a better estimate throughout and equals $d_{b}^{'}$ at large separation.

In higher dimensions, $d_{a}^{'}\leq d_{e}^{'}$ still, and they still usually underestimate $d_{b}^{'}$ (especially when means are close), but there are exceptions (Figure 4a, right, and Figure 2d).

We can theoretically show that $d_{a}^{'}\leq d_{e}^{'}$ in all dimensions and cases. In 1d, this is simply because $\sigma_{\text{avg}}\leq \sigma_{\text{rms}}$, and at the limit of highly unequal sds, $\sigma_{\text{avg}}/\sigma_{\text{rms}}\to 1/\sqrt{2}$, so $d_{a}^{'}\to d_{e}^{'}/\sqrt{2}$, which is the 30% underestimate. In higher dimensions, we can show analogous results using Figure 4b as an example. The left figure shows two normals with error ellipses corresponding to their sds, and their average and root-mean-square (rms) sds. Now we make two linear transformations of the space: First we standardize normal a, then we diagonalize normal b (i.e., a rotation that aligns the axes of error ellipse b with the coordinate axes). In this space (right figure), $S_{a}=1$, $S_{b}$ is diagonal, and the axes of $S_{\text{avg}}$ and $S_{\text{rms}}$ are the average and rms of the corresponding axes of $S_{a}$ and $S_{b}$. $S_{\text{rms}}$ is hence bigger than $S_{\text{avg}}$, so has larger overlap at the same separation, so $d_{a}^{'}\leq d_{e}^{'}$. The ratio of $d_{a}^{'}$ and $d_{e}^{'}$ is $∥S_{\text{rms}}^{-1}\mu ∥/∥S_{\text{avg}}^{-1}\mu ∥$, the ratio of the 1d slices of the average and rms sds along the axis through the means. When these are highly unequal, we again have $d_{a}^{'}\to d_{e}^{'}/\sqrt{2}$ in general dimensions.

We can also show that at large separation in 1d, $d_{e}^{'}$ converges to $d_{b}^{'}$. Consider normals at 0 and 1 with sds sσ and σ (Figure 4c). At large separation (σ→0), the boundary points, where the distributions cross, are $\frac{s}{s\pm 1}$. The right boundary is $\frac{1}{\sigma (s-1)}$ sds from each normal, so it adds as much accuracy for the left normal as it subtracts for the right. So only the inner boundary is useful, which is $\frac{1}{\sigma (s+1)}$ sds from each normal. The overlap here thus corresponds to $d_{b}^{'}=\frac{2}{\sigma (s+1)}=d_{e}^{'}$. So, when two 1d normals are too far apart to compute their overlap (see performance section) and hence $d_{b}^{'}$, the toolbox returns $d_{e}^{'}$ instead.

Given that $d_{e}^{'}$ is often the better approximation to the best discriminability $d_{b}^{'}$, why is $d_{a}^{'}$ used so often? Simpson and Fitter (1973) argued that $d_{a}^{'}$ is the best index, because it is the accuracy in a two-interval task with stimuli $x_{1}$ and $x_{2}$, using the criterion $x_{1}>x_{2}$. But as we saw, this is not the optimal way to do this task. The optimal error p(e) is instead as calculated previously, and $d_{b}^{'}\left(ab,ba\right)=2Z\left(1-p\left(e\right)\right)$ is the best discriminability. Unfortunately, this does not have a simple relationship with $d_{b}^{'}\left(a,b\right)$ for the yes/no task. But we can calculate mathematically here that $d_{e}^{'}\left(ab,ba\right)=\sqrt{2}d_{e}^{'}\left(a,b\right)$, which may still better approximate the best discriminability than $d_{a}^{'}\left(ab,ba\right)=\sqrt{2}d_{a}^{'}\left(a,b\right)$.

A brief note about Grey and Morgan's (1972) approximate index, which uses the geometric mean of the sds: This behaves inconsistently; it underestimates $d_{b}^{'}$ at small discriminability, but overestimates it at large discriminability.

In sum, $d_{b}^{'}$ is the maximum discriminability between normals in all cases, including two-interval tasks, especially when means are closer and variances are unequal. $d_{e}^{'}$ often approximates it better than $d_{a}^{'}$ (e.g., when the decision variable in a classification task is modeled as two unequal-variance 1d normals).

#### Receiver operating characteristic (ROC) curves

**Figure 5.**
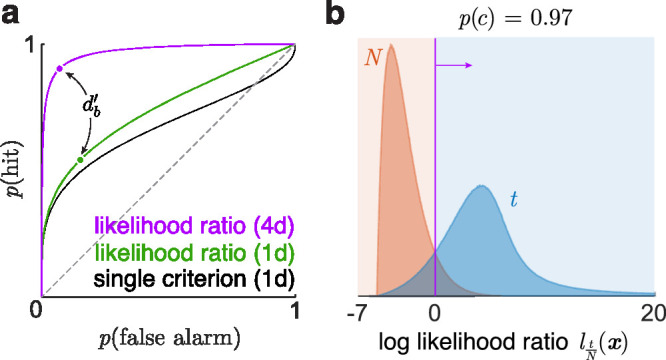
Receiver operating characteristic (ROC) curves. (**a**) Yes/no ROC curves for a single shifting criterion (black) versus a shifting likelihood ratio (green) between the two 1d normals of Figure 3a (adapted from Wickens, 2002, fig. 9.3) and a shifting likelihood ratio between a normal and a t distribution in 4d (purple). The optimal two-interval accuracies of the 1d normals (Figure 3c) and the 4d distributions (Figure 5b) are 0.74 and 0.97, equal to the areas under their likelihood ratio curves here. The points marked on these curves are the farthest from the diagonal and correspond to the Bayes discriminability. (**b**) Distributions of the log likelihood ratio of the 4d t versus normal distribution. Sweeping the criterion corresponds to moving along the purple ROC curve of a.

ROC curves track the outcome rates from a single criterion swept across two 1d distributions (e.g., black curve of Figure 5a) or varying the likelihood ratio between any two distributions in any dimension (green and purple curves), which corresponds to sweeping a single criterion across the 1d distributions of the likelihood ratio l (Figures 3b and 5b for the green and purple curves).

Discriminability indices are frequently estimated from ROC curves. $d_{a}^{'}$ is $\sqrt{2}$ times the z-score of the single-criterion ROC curve area. $d_{b}^{'}$ has no such simple relationship with curve area but can be estimated in different ways. Even though $d_{b}^{'}$ uses both criteria for unequal-variance 1d normals, it can still be estimated from the usual single-criterion ROC curve. Assume that the normals are N(0,1) and N(μ,σ). From the single-criterion ROC curve, we first estimate μ and σ, and then we use our method to compute $d_{b}^{'}$ of normals with these parameters.

$d_{b}^{'}$ can also be estimated from a likelihood ratio ROC curve. For any two distributions in any dimensions, $d_{b}^{'}$ corresponds to the accuracy at the point along their likelihood ratio ROC curve that maximizes p(hit)-p(falsealarm), which is the farthest point from the diagonal, where the curve tangent is parallel to the diagonal (Figure 5a).

#### Custom classifiers

Sometimes, instead of the optimal classifier, we need to test and compare suboptimal classifiers, for example, one that ignores a cue, or some cue covariances, or a simple linear classifier. So the toolbox allows the user to extract the optimal boundary and change it, and explicitly supply some custom suboptimal classification boundary. Figure 2d compares the classification of two bivariate normals using the optimal boundary (which corresponds to $d_{b}^{'}$) versus using a hand-supplied linear boundary. Just as with integration, one can supply these custom classification domains in quadratic, ray-trace, or implicit form, and use set operations on them.

### Classifying using data

If instead of normal parameters, we have labeled data as input, we can estimate the parameters. The maximum-likelihood estimates of means, covariances, and priors of normals are simply the sample means, covariances, and relative frequencies. With these parameters, we can compute the optimal classifier β(x) and the error matrix. We can further calculate another quadratic boundary γ(x) to better separate the given samples: Starting with β(x), we optimize its (k+1)(k+2)/2 independent parameters to maximize the classification outcome value of the given samples. This is important for non-normal samples, where the optimal boundary between estimated normals may not be a good classifier. This optimization then improves classification while still staying within the smooth quadratic family and preventing overfitting. Figure 2e shows classification based on labeled non-normal samples.

If, along with labeled samples, we supply a custom quadratic classifier, the toolbox instead optimizes this for the sample. This is useful, say, in the following case: Suppose we have already computed the optimal classifier for samples in some feature space. Now if we augment the data with additional features, we may start from the existing classifier (with its coefficients augmented with zeros in the new dimensions) to find the optimal classifier in the larger feature space.

### Multiple normals

The optimal classifier between two normals is a quadratic, so error rates can be computed using the generalized chi-square method or the ray-trace method. When classifying among more than two normals, the decision region for each normal is the intersection of its quadratic decision regions $q_{n}^{i}\left(x\right)>0$ with all the other normals i and may be written as:

$$\begin{array}{c} f\left(x\right)=\min_{i}q_{n}^{i}\left(x\right)>0. \end{array}$$

This is not a quadratic, so only the ray-trace method can compute the error rates here by using the intersection operation on the domains as described before. Figure 2f shows the classification of several normals with arbitrary means and covariances.

### Combining and reducing dimensions

It is often useful to combine the multiple dimensions in a problem to fewer or one dimension (Oruç et al., 2003). Mapping many-dimensional integration and classification problems to fewer dimensions allows visualization, which can help us understand multivariate normal models and their predictions, and to check how adequately they represent the empirical or other theoretical probability distributions for a problem.

As we have described, the multidimensional problem of integrating a normal probability in the domain f(x)>0 can be viewed as the 1d integral of the pdf of f(x) above 0. Similarly, multidimensional binary classification problems with a classifier f(x) can be mapped to a 1d classification between two distributions of the scalar decision variable f(x), with the criterion at 0, while preserving all classification errors. For optimally classifying between two normals, mapping to the Bayes decision variable β(x) is the optimal quadratic combination of the dimensions. For integration and binary classification problems in any dimensions, the toolbox can plot these 1d “function probability” views (Figure 2g). With multiple classes, there is no single decision variable to map the space to, but the toolbox can plot the projection along any chosen vector direction. Figure 2h shows the classification of samples from four 4d t distributions using normal fits, projected onto the axis along (1,1,1,1).

For a many-dimensional classification problem, we can also define a decision variable on a subset of dimensions to combine them into one, then combine those combinations further and so on, according to the logic of the problem.

In the sections below, we shall see examples of such applications, where we map to fewer dimensions to see how well a multivariate normal model works for a problem, and also combine groups of cues to organize a problem and get visual insight.

### Testing a normal model for classification

The results developed here are for normal distributions. But even when the variables in a classification problem are not exactly normal (e.g., either they are an empirical sample, or they are from some known but non-normal distribution), we can still use the current methods if we check whether normals are an adequate model for them. One test, as described before, is to project the distributions to one dimension, either by mapping to a quadratic form (Figure 2g) or to an axis (Figure 2h), where we can visually compare the projections of the observed distributions and those of their fitted normals.

We could further explicitly test the normality of the variables with measures like negentropy, but this is stricter than necessary. If the final test of the normal model is against outcomes of a limited-trials classification experiment, then it is enough to check for agreement between outcome counts predicted by the true distributions and their normal approximations, given the number of trials. For any classification boundary, we can calculate outcome rates, for example, p(A|a) for a hit, determined from the true distributions versus from the normal approximations. The count of hits in a task is binomial with parameters equal to the number of a trials and p(A|a), so we can compare its count distribution between the true and the normal model.

If the classes are well-separated (e.g., for ideal observers), the optimal boundary provides near-perfect accuracy on both the true and the normal distributions, so comparing yields no insight. To make the test more informative, we repeat it as we sweep the boundary across the space into regions of error, to show if the normal model still stands. This is similar to how the decision criterion between two 1d distributions is swept to create an ROC curve that characterizes the classification more richly than a single boundary. In multiple dimensions, there is more than one unique way to sweep the boundary. We pick two common suboptimal boundary families. The first corresponds to an observer being biased toward one type of error or another (i.e., a change in the assumed ratio of priors or outcome values). The second is an observer having an internal discriminability different from the true (external) one (e.g., due to blurring the distributions by internal noise), so adopting a boundary corresponding to covariance matrices that are scaled by a factor. When there are two classes, the boundaries for both of these suboptimal observers correspond to a shift in the constant offset $q_{0}$ (Equation 3), that is, a shift in the likelihood ratio of the two normals. So we are simply moving along the normal likelihood ratio ROC curves, as we compare the outcome rates of the true and the normal distributions.

**Figure 6.**
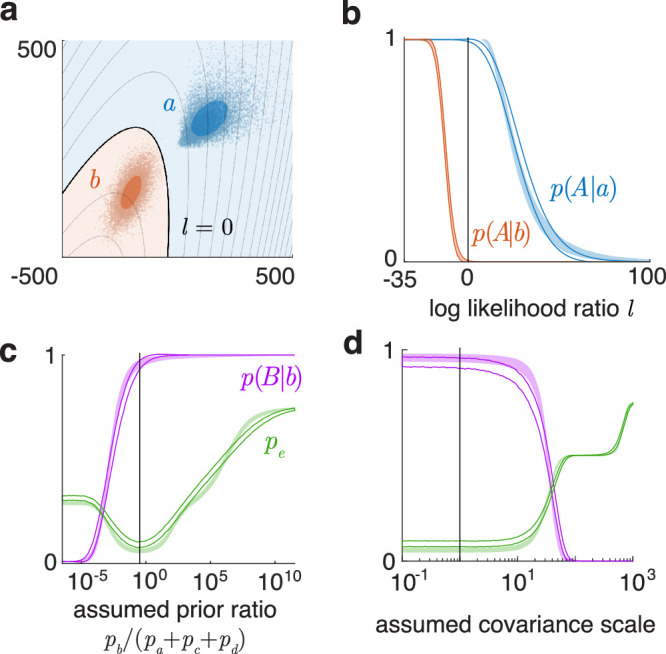
Testing normal approximations for classification. (**a**) Classifying two empirical distributions (a is not normal). Gray curves are contours of l, that is, the family of boundaries corresponding to varying likelihood ratios of the two fitted normals. (**b**) Mean ± sd of hit and false alarm fractions observed (color fills) versus predicted by the normal model (outlines), along this family of boundaries. Vertical line is the optimal boundary. (**c**) Similar bands for class b hits and overall error, for the 4d four-class problem of Figure 2h, across boundaries assuming different priors $p_{b}$ and (**d**) across boundaries assuming different covariance scales ($d^{'}$s).

Figure 6a shows the classification of two empirical distributions, where a is not normal, and gray curves show this family of boundaries, which are simply contours of the log likelihood ratio l. Since a and b are well-separated, the ROC curves for both true and normal distributions would hug the top and left margins, so they cannot be compared. Instead, we detach the hits and false alarms from each other and plot them individually against the changing likelihood ratio criterion, which gives us more insight. Figure 6b shows the mean ± sd bands of hits and false alarms from applying these boundaries on samples of 100 trials (typical of a psychophysics experiment) from each true distribution versus the normal approximations. They exactly coincide for false alarms/correct rejections but deviate for hits/misses, correctly reflecting that b is normal but a is not. The investigator can judge if this deviation is small enough to be ignored for their problem.

Now consider the case of applying these tests to multiclass problems. The two kinds of suboptimal boundaries we picked are no longer the same family here. Recall that the classification problem of 2h had four 4d t distributions. Figure 6c shows similar tests to see if this problem (with priors now equal) is well-modeled by normals. The family of boundaries corresponds to varying the assumed prior $p_{b}$. We may compare any of the 16 outcome rates here, for example, p(B|b), and also the overall error p(e). When there are multiple classes, for any given true class, the numbers of responses in the different classes are multinomially distributed, so that the total number of wrong responses is again binomially distributed. p(e) is the prior-weighted sum of these binomially distributed individual errors, so we can calculate its mean and sd predicted by the observed versus the normal distributions. Figure 6d shows the test across boundaries corresponding to all covariance matrices scaled by a factor, changing the $d^{'}$ between the classes.

Some other notable suboptimal boundaries to consider for this test are ones that correspond to adding independent noise to the cues (which changes only their variances but not their covariances), ones that ignore certain cues or cue covariances, or simple flat boundaries. As seen here, even for many-dimensional distributions that cannot be visualized, these tests can be performed to reveal some of their structure and to show which specific outcomes deviate from normal prediction for which boundaries.

When the problem variables have a known non-normal theoretical distribution, the maximum-likelihood normal model is the one that matches its mean and covariance, and these tests can be performed by theoretically calculating or bootstrap sampling the error rate distributions induced by the known true distributions.

## MATLAB toolbox: Functions and examples

For an integration problem, the toolbox provides a function that inputs the normal parameters and the integration domain (as quadratic coefficients or a ray-trace or implicit function) and outputs the integral and its complement, the boundary points computed, and a plot of the normal probability or function probability view. The function for a classification problem inputs normal parameters, priors, outcome values, and an optional classification boundary; outputs the coefficients of the quadratic boundary β and points on it, the error matrix, and discriminability indices $d_{b}^{'}$, $d_{a}^{'}$ and $d_{e}^{'}$; and produces a normal probability or function probability plot. With sample input, it additionally returns the coefficients of γ and points on it, error matrices and $d_{b}^{'}$ values corresponding to classification accuracies of the samples using β and γ, and the mapped scalar decision variables β(x) and γ(x) from the samples. The toolbox also provides functions to compute pdfs, cdfs, and inverse cdfs of functions of normals.

**Figure 7.**
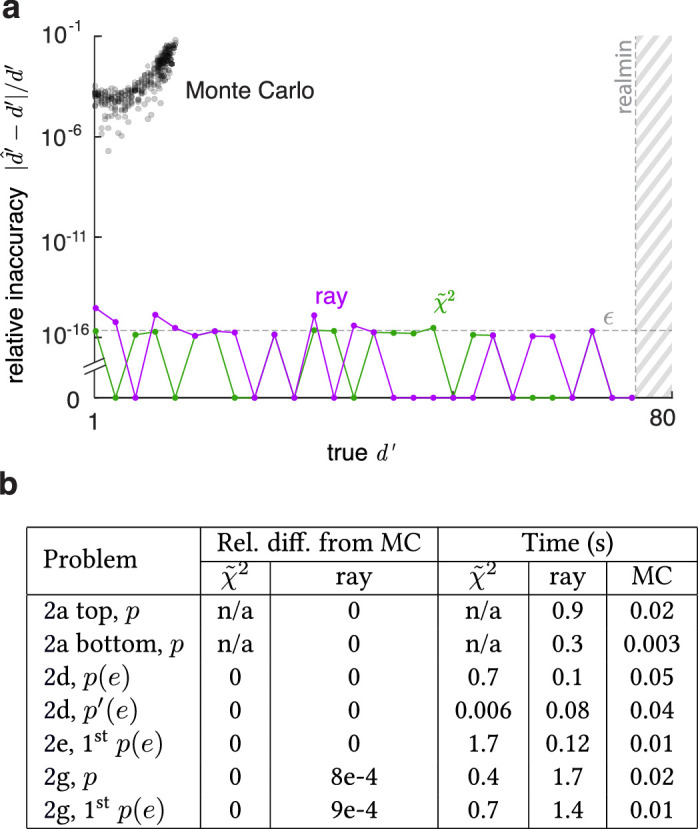
Performance benchmarks of the generalized chi-square (denoted by ${\tilde{\chi }}^{2}$) and ray-trace methods, against a standard Monte Carlo method. (**a**) Relative inaccuracies in $d^{'}$ estimates by the Monte Carlo method (across multiple runs), and our two methods, as the true $d^{'}$ increases. Monte Carlo estimates that take similar time rapidly become erroneous, failing beyond $d^{'}\approx 10$. Our methods stay extremely accurate (around machine epsilon ε) up to $d^{'}\approx 75$, which corresponds to the smallest error rate representable in double precision (“realmin”). (**b**) For several problems of Figure 2, relative differences in the outputs of the two methods from the Monte Carlo estimate and computation times for 1% precision.

**Figure 8.**
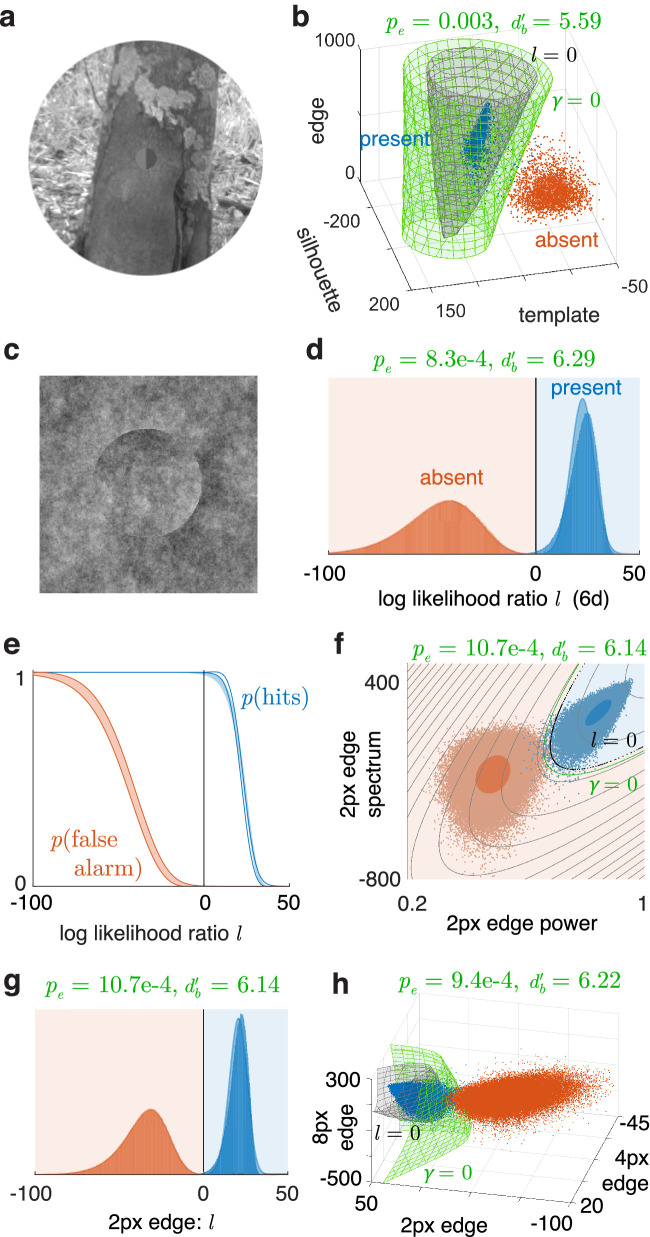
Applying the method and toolbox to visual target detection studies. (**a**) Example image of a target on a natural background. (**b**) Classification of images with the target present or absent, in the space of three cues. p(e) and $d_{b}^{'}$ correspond to the classification error of the sample points using γ. (**c**) Example image for camouflage detection. (**d**) Classifying these using six cues, viewed in terms of the log likelihood ratio l. (**e**) Bootstrap mean ± sd of hit and false alarm fractions from applying a family of boundaries (corresponding to varying the criterion likelihood ratio) on 100 samples of the true 6d cue distributions (color fills) versus their normal approximations (outlines). (**f**) Classifying with only two cues computed at 2px. Gray curves are contours of the log-likelihood ratio l. (**g**) Combining the two cues of plot f into one using l (i.e., the space of plot f has been projected along the gray contours). (**h**) Classifying with such combined cues at three scales.

Many different example problems, including every problem discussed in this article (examples in Figures 2, 3, 4, and 5; tests in Figures 6 and 7; and research applications in Figure 8) are available as interactive demos in the “getting started” live script of the toolbox and can be easily adapted to other problems.

## Performance benchmarks

In this section, we test the performance (accuracy and speed) of our MATLAB implementations of the generalized chi-square and ray-trace algorithms against a standard Monte Carlo integration algorithm. We first set up a case where we know the ground truth and compare the estimates of all three methods at the limit of high discriminability (low error rates) where it is most challenging (which occurs for computational models and ideal observers). We take two 3d normals with the same covariance matrix, so that the true discriminability $d^{'}$ is exactly calculated as their Mahalanobis distance. Now we increase their separation while computing the optimal error with each of our methods at maximum precision and a discriminability from it. The generalized chi-square method is very fast (due to the trivial planar boundary here), and the ray-trace method takes an average of 40 s. For a fair comparison, we use $10^{8}$ samples for the Monte Carlo, which also takes ∼40 s. Each method returns an estimate ${\hat{d}}^{'}$. Figure 7a shows the relative inaccuracies $|{\hat{d}}^{'}-d^{'}|/d^{'}$ as true $d^{'}$ increases. With increasing separation, the Monte Carlo method quickly becomes inaccurate, since the error rate (i.e., the probability content in the integration domain) becomes too small to be sampled. The method stops working beyond $d^{'}\approx 10$, where none of the $10^{8}$ samples fall in the error domain. In contrast, inaccuracies in our methods are extremely small, of the order of the double-precision machine epsilon ε, demonstrating that the algorithms contain no systematic error besides machine imprecision (however, MATLAB's native integration methods may not always reach the desired precision for a problem). This is possible because a variety of techniques are built into our algorithms to preserve accuracy, such as holding tiny and large summands separate to prevent rounding, using symbolic instead of numerical calculations, and using accurate tail probabilities. The inaccuracies do not grow with increasing separation, until $d^{'}\approx 75$, which corresponds to the smallest error rate p(e) representable in double precision (“realmin” = 2e-308), beyond which both methods return p(e)=0 and $d_{b}^{'}=\infty$. For 1d problems beyond this, the toolbox returns $d_{e}^{'}$ instead.

Next, we compare the three methods across several problems of Figure 2. The values here are large enough that Monte Carlo estimates are reliable and quick, so we use it as a provisional ground truth. We compute the values with all three methods up to maximum practicable precisions, then calculate the relative (fractional) differences of our methods from the Monte Carlo. If a value is within the spread of the Monte Carlo estimates, we call the relative difference 0. Figure 7b lists these, along with the times to compute the values to 1% precision on an AMD Ryzen Threadripper 2950X 16-core processor. We see that both of our methods produce accurate values at comparable speeds.

## Applications in visual detection

We demonstrate the use of these methods in visual detection tasks that have multiple cues with different variances and correlations.

### Detecting targets in natural scenes

We have applied this method in a study to measure how humans compare against a nearly ideal observer in detecting occluding targets against natural scene backgrounds in a variety of conditions (Walshe & Geisler, 2020). We placed a target on a random subset of natural images, then blurred and downsampled them to mimic the effect of the early visual system (Figure 8a). We sought to measure how well the targets on these degraded images can be detected using three cues: related to the luminance in the target region, the target pattern, and the target boundary. We computed these cues on the set of images. They form two approximately trivariate normal distributions for the target-present and target-absent categories. We then computed the decision boundary, error rate, and $d_{b}^{'}$ against varying conditions. Figure 8b shows the result for one condition, with a hyperboloidal boundary. These error rates and $d_{b}^{'}$s can then be compared across conditions.

### Detecting camouflage

We also applied this method in a study measuring performance in detecting camouflaged objects (Das & Geisler, 2018). The major cue for detecting the object (Figure 8c) is its edge, which we compute at scales of 2px, 4px, and 8px. We extract two scalar features from the edge at each scale: The edge power captures its overall prominence, and the edge spectrum characterizes how this prominence is distributed along the boundary. We thus have six total features. Figure 8d shows the classification of these images using these six features, projected onto the Bayes decision variable (log-likelihood ratio) l. In this reduced dimension, we can see that the absent distribution is quite normal, and present is nearly so. Consistently, in a normality test for classification with 100 trials, Figure 8e, the hit fraction deviates only marginally from its normal prediction, so we accept the normal model here. Figure 8f shows classification using only the 2px features. We use our dimension reduction technique to combine these two cues into the Bayes decision variable l of this space, which we call simply the 2px edge cue. Classifying using this single variable, Figure 8g, is the same as the 2d classification of Figure 8f and preserves the errors. We do the same merging at 4px and 8px, thus mapping six features to three. Figure 8h shows the classification using these three merged cues. Due to the information in the two added scales, the classification has improved. The total number of classifier parameters used in this sequential classification is 28 (6 for each of the three 2d classifiers, then 10 when combining them in 3d). The classifier in full 6d has 28 parameters as well, yet it performs better since it can simultaneously optimize them all. Even so, merging features allows one to combine them in groups and sequences according to the problem structure and visualize them.

## Conclusions

In this article, we presented our methods and open-source software for computing integrals and classification performance for normal distributions over a wide range of situations.

We began by describing how to integrate any multinormal distribution in a quadratic domain using the generalized chi-square method, then presented our ray-trace method to integrate in any domain, using examples from our software. We explained how this is synonymous with computing cdfs of quadratic and arbitrary functions of normal vectors, which can then be used to compute their pdfs and inverse cdfs as well.

We then described how to compute, given the parameters of multiple multinormals or labeled data from them, the classification error matrix, with optimal or suboptimal classifiers, and the maximum (Bayes-optimal) discriminability index $d_{b}^{'}$ between two normals. We showed that the common indices $d_{a}^{'}$ and $d_{e}^{'}$ underestimate this, and that contrary to common use, $d_{e}^{'}$ is often a better approximation than $d_{a}^{'}$, even for two-interval tasks.

We next described methods to merge and reduce dimensions for normal integration and classification problems without losing information. We presented tests for how reliably all the above methods, which assume normal distributions, can be used for other distributions. We followed this by demonstrating the speed and accuracy of the methods and software on different problems.

Finally, we illustrated all of the above methods on two visual detection research projects from our laboratory.

Although not developed here, the approach of our ray-trace integration method may carry over to other univariate and multivariate distributions. In the method, we spherically symmetrize the normal, find its distribution along any ray from the center, and then add it over a grid of angles. This transforms all problem shapes to the canonical spherical form, then efficiently integrates outward from the center of the distribution. Some distributions (e.g., lognormal) can simply be transformed to a normal and then integrated with this method. For example, if y∼lognormal(μ=1,σ=0.5), then we can compute, say, $p\left(\sin y>0\right)=p\left(\sin e^{x}>0\right)=0.65$ (where x is normal) and all other quantities such as pdfs, cdfs, and inverse cdfs of its arbitrary functions (see toolbox example guide).

For other distributions, our general method is still useful if they are already spherically symmetric (i.e., spherical distributions), or can be made so (e.g., elliptical distributions), and the ray distribution through the sphere can be found. When they cannot be spherized, the ray distribution (if calculable) will depend on the orientation, just as the integration domain does. But once this additional dependency has been taken into account, integrating along rays from the center should still be the efficient method for distributions that fall off away from their center.
